# An Innovative Application of High-Fidelity Medical Simulators to Objectively Demonstrate the Impact of Sports on the Development of Fine Motor Skills—A Pilot Study

**DOI:** 10.3390/s25175316

**Published:** 2025-08-27

**Authors:** Peter Szikra, Adam Attila Matrai, Adam Varga, Laszlo Balogh, Zoltan Karacsonyi, Konrad Okros, Tamas Horovitz, Miklos Toth, Norbert Nemeth

**Affiliations:** 1 Department of Health Sciences and Sport Medicine, Institute for Sports and Health Sciences, Hungarian University of Sports Science, Alkotas u. 42-48, H-1123 Budapest, Hungary; szikra.peter@tf.hu (P.S.); toth.miklos@tf.hu (M.T.); 2 Department of Operative Techniques and Surgical Research, Faculty of Medicine, University of Debrecen, Moricz Zsigmond Str. 22, H-4032 Debrecen, Hungary; matrai.adam@med.unideb.hu (A.A.M.); varga.adam@med.unideb.hu (A.V.); 3 Department of Cardiology and Cardiac Surgery, Faculty of Medicine, University of Debrecen, Moricz Zsigmond Str. 22, H-4032 Debrecen, Hungary; balogh.laszlo@med.unideb.hu; 4 Department of Orthopedics Surgery and Traumatology, Faculty of Medicine, University of Debrecen, Nagyerdei Str. 98, H-4032 Debrecen, Hungary; karacsonyi.zoltan@med.unideb.hu (Z.K.); okros.konrad@med.unideb.hu (K.O.); 5 Table Tennis Club of Debrecen, University Athletics Club of Debrecen, Saletrom Str. 3, H-4025 Debrecen, Hungary; horovitz61@gmail.com

**Keywords:** manual skills, skill performance, skill assessment, sport training program, table tennis, high-fidelity simulators, medical education

## Abstract

**Highlights:**

**What are the main findings?**

- High-fidelity medical simulators with basic training modules can give useful feedback for fine motor skill development.
- Table tennis has been identified as a sport that can be used to maintain or improve arthroscopy and develop moderate catheter manipulation skills on simulators.

**What is the implication of the main finding?**

- Table tennis could improve fine motor skills that are important in good performance on high-fidelity medical simulators to prepare for the clinical practice.

**Abstract:**

Operative medicine needs fine manual skills; therefore, several educational training programs focus on skill development as well. Related to sports sciences, various sport types are also dependent on fine motor skills. We hypothesized that an adequate sport training program may contribute to the development of medical students’ manual dexterity. We conducted objectively tests using high-fidelity medical simulators. Volunteer medical students were delegated to table tennis group (TG), where students participated in 2 h/week of table tennis training for 7 weeks, or to a Control group (CG) that included students without regular sport activity. Objective data on fine motor skills during completion of basic modules of high-fidelity vascular catheterization and arthroscopy simulators were recorded before and after the 7-week period. In the TG group, significant differences were found in time and quality parameters compared to CG. On the vascular catheterization simulator basic navigation module, all time parameters improved. On the arthroscopy simulator basic skill module, the total performance and safety scores significantly improved, and procedure time decreased. In conclusion, high-fidelity vascular catheterization and arthroscopy medical simulators with basic training modules could provide useful feedback for fine motor skill development. The intensive table tennis training program was effective in maintaining/improving medical students’ fine manual skills.

## 1. Introduction

Manual skills and fine movements have great importance in many fields of medicine for successful and safe interventions [1,2,3,4]. Today, minimally invasive techniques such as arthroscopy or catheter-based interventional cardiology have a significant share in medicine, alongside open surgical techniques [5,6,7,8,9,10,11]. While in classical surgical care the surgeon sees and controls their own movement in three dimensions (3D), in scopic or catheter techniques, 3D movement must be controlled on a monitor representing two dimensions only. The arthroscopic and catheter manipulation technique also differs from the open surgical technique in that the magnified image on the monitor shows the instrumental manipulation with a greater degree of movement corresponding to the magnification compared to the movement of the hands.

In the practical medical professions, it is evident that the skill level of the operator influences the quality of care, and greater skill is associated with fewer postoperative complications [3,4]. The performance of the operating surgeon does not depend only on the development of their manual skills or spatial orientation; surgical performance may be more accurately explained through the combined effects of physical, cognitive, visual, and psychological variables [1,12,13]. In the field of bariatric surgery, John D. Birkmeyer and colleagues have demonstrated that greater skill is associated with fewer postoperative complications and lower rates of reoperation, readmission, and visits to the emergency department [3]. Manual dexterity and visual–spatial ability are considered key to the development of superior laparoscopic skills [1,14]. During the learning curve for laparoscopic totally extraperitoneal repair of inguinal hernia, the mean duration of surgery significantly decreased in relation to experience; authors estimate that 60 cases are needed for a beginner surgeon to prevent unnecessary complications and shorten the duration of surgery [4]. This implies that it is important that when the surgeon starts patient care, they must be as well prepared as possible to keep the operating time and complication rate as low as possible. Based on the latest research findings, it is proven that improvement in surgical education training will benefit patients by reducing medical errors [15].

Numerous educational training programs are known for skill development; many of them are part of medical education [16,17,18,19,20]. There are many attempts worldwide in which medical universities and medical device provider industries look for ways to support healthcare professionals in the high-level implementation of the practical implementation of operation techniques [20,21,22]. Progress in surgical education is primarily driven by the rapid evolution of simulator technologies and their high level of involvement in manual skill development training [23,24]. High-fidelity simulator technology explosively developing systems not only provides an opportunity for students and operators to practice but also provides a quantitative control opportunity to assess basic practical skills [23,25,26]. Simulator-based assessment of aptitude appears to have the potential to represent a job sample and enable the assessment of all forms of aptitude for laparoscopic surgery at once [24]. Arthroscopic surgery has always included complexities that require substantial hand–eye coordination, manual dexterity, and knowledge of anatomy. The Proficiency-Based Progression (PBP) model is an approach in which the trainee must acquire and demonstrate basic skill sets before progressing to more advanced techniques. Orthopedic residents randomized to the PBP curriculum completed significantly more tasks and enacted 55% fewer errors than comparable level residents who underwent traditional training [21].

In interventional cardiology, proper catheter use is essential; complications occur as a result of mechanical manipulation of the culprit lesion during cardiac intervention [27,28]. In addition to gentle guidewire and catheter use, rapid delivery of the catheter system to the site of stenosis or artery occlusion is also very important in case of acute coronary infarction treatment. Virtual reality simulation of cardiovascular procedures can contribute to surgical training and improve the educational experience without putting patients at risk [29,30]. After the simulation task procedure is completed, there are scoring systems that provide detailed technical feedback on the procedure, including unsafe catheter maneuvers and inappropriate wire placement. Simulator training has now been widely accepted as an important tool for procedural training and is seen as an important future development for interventional cardiology training programs across the world [31]. Continuous improvement of surgeons’ manual skills is important and requires the right amount and quality of practice [32].

In relation to sports sciences, fine motor skills are involved in many physical activities and are prerequisites for the performance of sport skills [33,34,35,36]. Whatever the sport, competitiveness requires increasingly difficult tasks and ever higher standards of execution. Thus, it seems useful to examine the effectiveness of the experience gained in practical development in sports for medical education.

We wished to investigate the question of whether table tennis experts can provide medical students with a developmental sports program that helps them improve their fine (arthroscopic and catheter manipulation) skills. We hypothesized that an appropriately chosen sport training program may help medical students to develop manual dexterity, which can be objectively tested on high-fidelity simulators. This study aimed to investigate the effectiveness of a table tennis training program on performance in high-fidelity arthroscopy and vascular catheterization simulators, requiring fine manual skills.

## 2. Materials and Methods

### 2.1. Participants and Study Design

The study was performed between October and December 2022 at the Faculty of Medicine of the University of Debrecen, Hungary. The study group included 26 volunteer medical students; the procedures followed were in accordance with the ethical standards (ethical committee approval: Regional Institutional Research Ethics Committee, Clinical Center, University of Debrecen (DE RKEB/IKEB: 6274-2022)) and the Helsinki Declaration of 1975, as revised in 2000.

Participants were assigned by personal agreement into two groups: CG without any exercise program (n = 13; 11 men and 2 women; age: 22 ± 1 years; BMI: 22.89 ± 2.29 kg/m^2^) and TG (n = 13; 11 men and 2 women; age: 22 ± 2 years, BMI: 23.46 ± 3.18 kg/m^2^). All the participating medical students had completed the ‘Basic Surgical Techniques’ subject [17] and had not performed any tasks on the simulators either before or during the period of skill level assessment and output skill level assessment exam. Participants could choose a group to join (initially named Group A and Group B, without knowing which would be the final Control or Training Group). In both groups, an equal number of seats were opened. After registration, we randomly selected (computer-generated order) which group would serve as the Control or Training Group.

All participants took a practical skills assessment at the beginning of the study, where the Mentice vascular catheterization simulator has been used with the Coronary Essentials module Navigation Training exercise (Mentice AB, Odinsgatan 10411 03 Gothenburg, Sweden) and a VirtaMed ArthroS™ arthroscopy simulator has been used with Triangulation Skills—Rings and Catch the Stars modules (VirtaMed AG. Rütistrasse 12, 8952 Schlieren, Zurich, Switzerland) (Figure S1).

In both groups, before completing the tasks, the participants filled in a questionnaire (Figure S2) and then watched an instructional video on the tasks to be completed.

They were then given the opportunity to try out the simulators technically, followed by the completion of the tasks. All study participants completed the skill level assessment test on the simulators.

After the skill level assessments, members of the TG participated in a 7-week table tennis training program (details in Section 2.2); the CG did not participate in any organized training that time. Participants did not engage in self-training outside of the two-hour weekly sessions. After the 7-week training program, all study participants attended the skill level assessment test on the simulators using the same method that has been applied as practical skills assessment at the beginning of the study. Study groups successfully completed both skill level assessment tests on simulators. None of the TG members missed more than 10% of the training sessions.

### 2.2. The Table Tennis Program

The dedicated table tennis training program (2 h per week for 7 weeks) was developed by the coach of the Debrecen University Athletics Club, Debrecen Table Tennis Club and Hungarian University of Sport Sciences working group member.

The table tennis exercises were designed according to the following key aspects: simultaneous use of two hands with different tasks for each hand, pair and group exercises, and temporarily breaking eye contact with the ball while performing tasks; as the study progressed, the exercises became more difficult. After the warm-up, special exercises were completed in the first half of the training session, the second half of the training session was a simple table tennis game (Table S1).

### 2.3. Skill Assessment on Simulators

#### 2.3.1. Vascular Catheter Simulator Exercise

The Navigation Training exercise of the Mentice vascular catheter simulator’s Coronary Essentials module aims to teach trainees the architecture of the coronary arteries and the skills necessary to successfully navigate the coronary system. Furthermore, it is critical to comprehend the treatment and selection of coronary intervention equipment.

Using a combination of software simulation technology and hardware sensors, the simulator recognizes and monitors the Navigation Training exercise. As access points for catheters and guidewires, the system has insertion sheaths that are fitted with sensors that can recognize the pushing, pulling, and rotating motions of the devices. These motions are converted into real-time navigation in a multi-screen virtual 3D coronary architecture, which offers accurate feedback from fluoroscopic and angiographic imaging. By simulating the resistance experienced during catheter manipulation using force-feedback technology, the simulator improves the tactile realism of the process.

The system continuously logs procedural parameters during Navigation Training, such as the duration of the entire process, the duration of the fluoroscopy, the quantity of contrast agent utilized, and the number of instrument changes (150 cm J-Curve standard guidewire, Judkins left/right 4.0 6F guide catheter, 150 cm 60-degree hydrophilic guidewire translation (cm) and rotation (turns)). With the utilization of these data, learning and skill evaluation are supported by prompt feedback and thorough procedure reports.

Objective evaluation of procedural skill and error detection are made possible by the simulator’s evaluation of technical performance and feedback on errors (Handling Events): injection in coronary arteries with a too steep angle, catheter scraping against vessel wall, catheter moving without wire support, catheter entered suboptimal vessel, selective catheter scraping against vessel wall, selective catheter moving without wire support, catheter too deep into ostium, selective catheter too deep into ostium, guide wire in small vessel, guide wire entered suboptimal vessel, 0.014 wire hit end of vessel, and 0.014 wire entered suboptimal vessel.

#### 2.3.2. Arthroscopy Simulator Exercise

Developing precise instrument handling and spatial coordination inside the joint space is the main goal of the Triangulation Skills Rings and Catch the Stars modules of the VirtaMed ArthroS simulator. The trainee must find virtual rings in the knee joint, insert the hook instrument into each ring, and keep it there for two seconds as part of the Triangulation Skills Rings module. To precisely target anatomical landmarks without causing harm, the user must learn to concurrently coordinate the camera and instrument, which enhances their bimanual dexterity and spatial vision.

The student utilizes a grasper to capture and remove virtual stars that are dispersed throughout the knee joint in the “Catch the Stars” module. The capacity to move items in a restricted joint environment, accurate tool control, and hand–eye coordination are all highlighted in this exercise.

By closely tracking the movement of the camera and instrument in the virtual joint environment, the simulator can identify the navigation process. The system provides real-time information on the location, orientation, and movement of the arthroscope and surgical instruments using sensors built into real-world devices that have been modified for virtual reality. To guarantee correct visualization and orientation within the joint, camera control and navigation monitor telescopic movements, picture centering, stability, and horizon alignment. The accuracy with which the trainee positions the instrument within the rings or grasps the stars is measured by the simulator, which also assesses how effectively the trainee coordinates the camera and the probe or grasper to find and interact with virtual objects (rings or stars). The program automatically verifies that tasks are being completed and, if dangerous maneuvers or cartilage contact are made, offers advice or severe error messages. To evaluate skill improvement, metrics like time spent, economy of movement, and camera and instrument path length are tracked.

Effective training on arthroscopic spatial orientation and bimanual coordination is made possible by the simulator’s ability to provide detailed, unbiased feedback on camera navigation, instrument handling, and triangulation skills during these modules through the combination of hardware tracking and software analysis.

**Figure 1:**
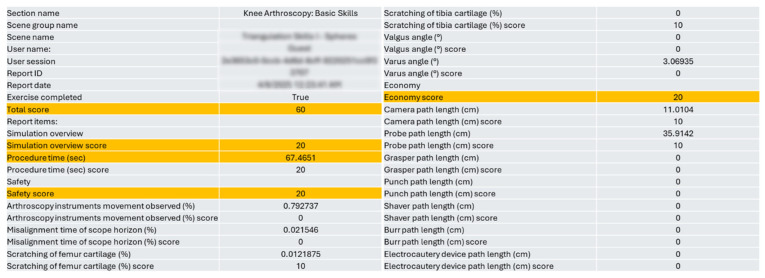
A representative chart of a completed practice on “Triangulation Skills: Rings and Catch the Stars” modules of the arthroscopy simulator.

Several important facets of arthroscopy skill performance are measured and reported by the simulator’s modules (Figure 1).

The developers of high-fidelity simulators worked with experienced arthroscopic and catheterization clinicians to determine the scores that form the basis for objective evaluation in the exam tasks. According to the manufacturer of the simulator, the “economy score” is the most efficient use of tools for quick but safe task completion, while the “safety score” estimates the risk of excessive force exerted on the tools during the procedure or force exerted on inadequate anatomical structures. Therefore, we considered these parameters useful and informative for our study.

The trainee’s ability to successfully coordinate the movement of the camera and instrument is demonstrated by the accuracy and precision of the placement of and interaction with virtual objects (such as rings or stars) within the joint space. The trainee’s ability to retain direction and visualization during the assignment is measured by camera navigation skills, which include steadiness, centering, horizon control, and length of camera movement routes. Instrument handling and triangulation assess two-hand coordination, the length and smoothness of an instrument’s movement, and the capacity to precisely position or grip things. The amount of time needed to finish tasks enables the evaluation of procedural speed and efficiency. The economy of movement is assessed to evaluate extraneous or superfluous camera and instrument movement. Error detection aids in the identification of technical errors and includes interaction with simulated cartilage or risky maneuvers.

### 2.4. Statistical Analysis

SigmaStat Software 3.1.1.0 (Systat Software Inc., San Jose, CA, USA) was used to carry out the statistical analyses. Data are presented as means ± S.D. (standard deviation). Depending on the result of the Kolmogorov–Smirnov normality tests, the first and second survey’s data were compared with a paired *t*-test or Wilcoxon rank sum test, while for analyzing the inter-group differences, Student’s *t*-test or Mann–Whitney rank sum test were used.

A *p*-value of <0.05 was considered statistically significant.

## 3. Results

### 3.1. Evaluation of the Questionnaire

Participants in both groups were of a similar age and had almost the same BMI values. The main anthropometric data are summarized in Table 1.

In the CG, one student played an instrument (ukulele), while in TG 3, students played an instrument (guitar or piano). The average time spent playing video games was 1 h per week in both groups (CG: four students, TG: five students). The time spent using the (smart) phone per day was almost the same in both groups (CG: 4.15 ± 1.28 h/day, TG: 4.17 ± 1.59 h/day). Handicraft hobbies were practiced by two people in the CG and three people in the TG. No one in the CG performed any regular sport activity, while five in the TG did. The most preferred form of transport in the CG was walking or driving, while in TG it was public transport. Weight training was performed by six people in both groups at the time of the survey. Other sports activities were few, the most common being running and football. Spiral tracking was rated on a scale of 1 to 3 points by two raters independently.

**Figure 2:**
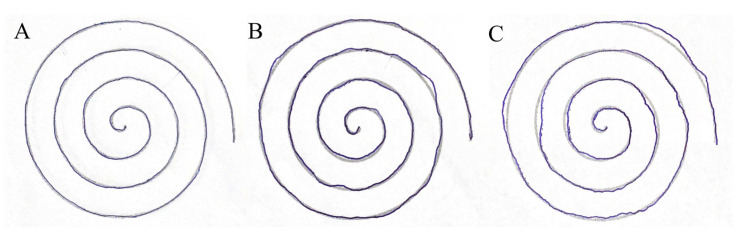
Representative examples on the performance scoring of free-hand spiral line tracking: (**A**) Three points, (**B**) two points, (**C**) one point.

Figure 2 shows the performance associated with each score on spiral line tracking (method: see Figure S2). In CG, the score was 2.57 ± 0.51 at the first survey and 2.69 ± 0.48 at the second survey. In TG, the values were 2.69 ± 0.6 and 2.93 ± 0.26 at the surveys, respectively.

### 3.2. Skill Assessment on Vascular Catheterization Simulator

For the CG, each point took longer to reach in the second survey than in the first survey (aortic root: *p* = 0.004; left main: *p* < 0.001). In TG, shorter times were needed to achieve some points, but not all (aortic root: *p* = 0.016). Overall, the time needed to reach the last point in the second survey was significantly less than the CG (right coronary artery: *p* = 0.038; distal right coronary artery: *p* = 0.048) (Table 2).

**Figure 3:**
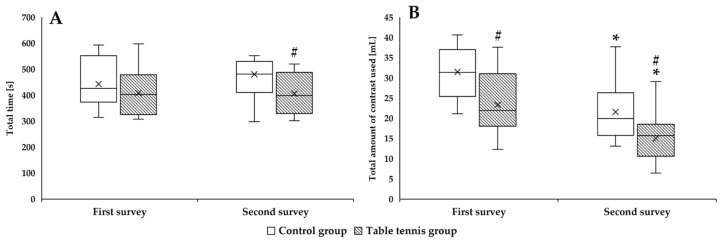
The time required for the vascular catheterization task (**A**) and the amount of contrast used (**B**) in the first and second surveys. Means ± S.D., * *p* < 0.05 vs. first survey (paired *t*-test/Wilcoxon rank sum test); ^#^ *p* < 0.05 vs. Control group (Student’s *t*-test/Mann–Whitney rank sum test).

Figure 3 shows the time needed to complete the task and the amount of contrast material used virtually. It can be observed that more time was needed to complete the task for the CG and less for the TG in the second survey. The score of the TG was significantly lower compared to the CG (*p* = 0.048). The amount of contrast agent used was significantly lower for both groups (CG: *p* = 0.003 vs. second survey; TG: *p* < 0.001 vs. second survey), but the TG used significantly less contrast than the CG in both assessments (first survey: *p* = 0.004; second survey: *p* = 0.016).

### 3.3. Skill Assessment on Arthroscopy Simulator

**Figure 4:**
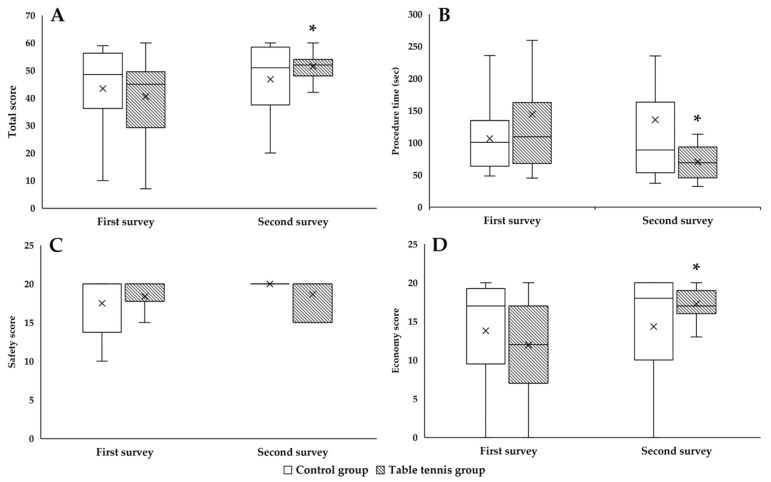
The total score (**A**) in the “Rings” module of the arthroscopy simulator, the time required for the procedure (**B**), and the safety score (**C**) and economy score (**D**). Means ± S.D., * *p* < 0.05 vs. first survey (paired *t*-test/Wilcoxon rank sum test).

In the “Rings” module of the arthroscopy simulator, the total score showed a significant improvement in the TG (*p* = 0.032) (Figure 4A). The time required for the intervention was significantly less in the TG in the second survey compared to the first survey (*p* = 0.032), while a slight increase was observed in the CG (Figure 4B). The safety score showed an improvement in both groups, although it was higher in the CG (Figure 4C). The economy score, which measures the movement of the inserted devices, was significantly higher in the TG (*p* = 0.013), suggesting that the TG made less unnecessary movement of the devices in the knee joint, thereby causing less abrasion of the anatomical structures (Figure 4D).

**Figure 5:**
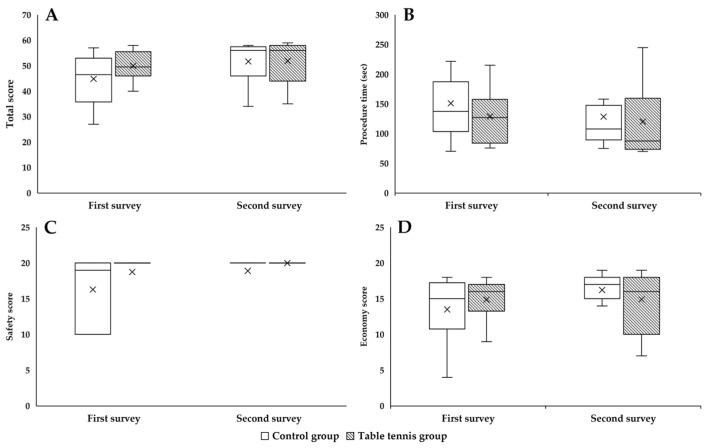
The total score (**A**) in the “Catch the Stars” module of the arthroscopy simulator, the time required for the procedure (**B**), the safety score (**C**), and the economy score (**D**). Means ± S.D.

Using the “Catch the Stars” module, no significant differences were observed in the total score, the time of intervention and the economy score in the second survey, although some improvement was also observed for this module. With regard to the safety score, it should be noted that all students in the TG scored at the maximum (Figure 5).

## 4. Discussion

It has been known for decades that the use of new technologies and related techniques gives patients a better chance of fast recovery and, on the other hand, the increasingly influential health insurers can save money preferring interventions with lower complication rates and shorter hospitalization times [37]. Minimally Invasive Surgical techniques have revolutionized the field of surgery, offering significant benefits over traditional open surgery [38].

The Hungarian University of Sports Sciences (HUSS) set up a scientific working group including sport specialists and technical trainers experienced in arthroscopy and interventional cardiology. The working group members analyzed arthroscopic and interventional cardiology operations performed by experienced surgeons. During the operations, the typical wrist and arm positions, optimal postures, movement characteristics and correct grip of the instruments were studied and identified. Based on the results, table tennis, badminton, fencing, shooting, and rhythmic gymnastics have been selected as sports where the typical operation movements are close to the arthroscopy and catheter-related operational techniques. The scientific team from the University of Debrecen—consisting of experts in interventional cardiology, arthroscopic surgery, and medical skill lab specialists—selected the table tennis as the most suitable sport from the list knowing local opportunities and conditions. Based on the results of Ying Gu and his team, table tennis physical activity can promote gross motor skills development, especially object control skills, so we thought it worth exploring the possibility of developing fine motor skills [39].

Surgeons performing arthroscopic or catheter manipulation have to occasionally take their eyes off the monitor to look at surgical instruments, another monitor, radiological images, or for other reasons. In skill labs, during exercises on simple task trainers or high-fidelity simulators, we have observed that when medical students take their eyes off the monitor, in many cases, they find it difficult to return to their original interpretation of the situation. We aimed to develop and accelerate this skill with these exercises as well.

The accurate application of several operating techniques and the proper use of different types of guidewires are the basis for complication-free interventional cardiology care [40]. The latest generation radiology imaging techniques have radically reduced the number of diagnostic arthroscopy and coronarography procedures [37,41]. These days, the indications for interventions are increasingly based on CT and MRI examinations; only preoperative diagnosis validation and the treatment provide opportunities to practice [42,43]. With a reduction in invasive diagnostic procedures, there are fewer opportunities to develop fine motor skills and learn how to use operating devices. We strongly believe it is important to look for interdisciplinary opportunities to develop practical skills. We can see various studies to explore how activities performed outside of work can help in the development of psychomotor and practical skills.

Andrea Moglia and colleagues monitored the positive effects of extracurricular activities on the psychomotor abilities of medical students, but a low correlation has been found between simulator scores and extracurricular activities, like videogames and musical instruments [23]. Fortunately, there is the interest of medical device manufacturers in ensuring that their operating systems give the best possible intervention results, which requires an interventionalist talented in fine motor skills and using their technology accurately and skillfully. These companies are also working on dedicated product and therapy training in cooperation with key opinion leader doctors, which usually includes practical skill development components. It is increasingly common to encounter forward-looking initiatives where operating device manufacturer companies, simulator companies and professional organizations collaborate to improve the quality of education, as can be seen in the case of the Arthroscopy Association of North America leaded program [21].

It can be established that exercises on a virtual simulator are a valid test of innate manual dexterity and can be considered to complement the selection process for a surgical training program, primarily to identify individuals with low innate aptitude for surgery [23]. However, Anetzberger and colleagues are aware that only enough simulator practice gives medical students a meaningful opportunity to develop [32]. It should be noted, however, that high-fidelity simulators are very expensive to buy and maintain. The investment required to acquire simulation hardware is significant, and there has been a demand from training bodies to see demonstrable benefits from simulator training before purchasing the devices, some costing more than GBP 90,000 [31]. According to Péter Tajti and colleagues, it is unclear at present whether the anticipated benefits exceed the associated risks and costs of simulation training of cardiac catheterization [44]. Currently, medical skill labs in medical education are staffed primarily by highly skilled doctors and technicians to lead and support practical training. Furthermore, it is a general experience that practicing on a high-fidelity simulator has a positive effect on medical students’ self-confidence.

We are not aware of any medical university that specifically employs physical education professionals to lead or support the movement and fine motor skills development of medical students, while the maneuvers applied in operations are increasingly complex and complicated. Even with a relatively low number of training sessions, table tennis sport presented promising results in manual skill development in this study. The sport specialists in the working group were not surprised that table tennis exercises resulted in more dominant improvements in arthroscopic performance, because these movements have a limited relationship with catheter manipulation. In interventional cardiology, the fine motor skills of the fingers play a more important role in moving the instruments than those of the wrist and arms, which are dominant in arthroscopy. According to the current study results and experiences in sport rhythmic gymnastics—including ribbon and ball exercises—it would be suitable as a sport for the development of catheter manipulation specific fine motor skills, but a separate study is needed to validate this. Results measured on both simulators show that a dedicated training program from sports professionals can be effective in improving the speed of intervention. The significant increase in economy score measured on an arthroscopy simulator not only affects the faster speed of operation observed in the previous score, but the economy movement also leads to a reduction in the fatigue of the intervener with all its positive benefits.

The residents’ performance in operations does not depend only on their fine motor skills; their medical knowledge, psychological state and mental abilities also play a decisive role in their performance. Initial results suggest that highly qualified sport trainers can identify the specific sports that best fit the practical skill development of the given medical discipline. A good coach can develop the students with such exercises that they improve their skills necessary for interventions in a playful way, enjoying the sport and building a good relationship with fellow students during the training sessions. An additional advantage may be the significantly low cost of practical skill development using sports compared to the use of expensive simulators but keeping the simulator exam function for monitoring the development of fine motor skills. It was a gratifying result of the research that we saw the medical students playing sports regularly with all kinds of benefits.

Targeted sports skill development for medical students to improve their manual skills using high-fidelity simulator testing accepted by the medical profession is currently a unique approach. In addition to the standard practical skills development exercises and programs currently used in medical training, we can also observe the exploration of innovative possibilities, including the role of playing musical instruments in fine motor skills. Musical tasks involve complex cerebral activity and ambidextrousity, which may have a positive impact on the acquisition of surgical skill sets. Sun and colleagues concluded that musical background is associated with better performance of fundamental surgical skills among surgical novices, particularly technique quality [45]. Comeau and colleagues were interested in whether musicians can learn and perform surgical skills more rapidly than non-musicians. Participants with piano expertise and participants with no formal music training learned how to perform a surgical knot and use sutures. Results for each group (musicians and non-musicians) were analyzed and compared, and musician participants finally performed the surgical tasks faster and received higher scores than the controls; for knot tying, the difference between the two groups was statistically significant [46]. Playing a musical instrument is a promising opportunity for medical students to develop their manual skills. Although we do not yet know how accurate this alternative technique is on a high-fidelity simulator control, it has shown initial success in simpler basic surgical task-trainer procedures. Of note is the study by Rui et al., who aimed at identifying expert musicians’ practice and performance strategies that may aid surgeons in enhancing their surgical performance. Based on a review of 82 relevant scientific articles, it was concluded that critical parallels exist between surgical and expert musical performance that may improve surgical outcomes by adopting musicians’ strategies for combating physiological and psychologic performance-related issues [47].

The limitations of this study are that the use of sports science training methods in this study lacks the possibility of individualized training, optimal adjustment of load magnitude, frequency and duration. Data decentralization and development of analysis methods linking related data input (e.g., from simulators) would also be useful in the future [48]. Another limitation is the relatively low case number, originating from the volunteer-based characteristic of the study, and from the burden on the active Table Tennis Club, the high utilization of simulators in undergraduate/graduate medical training, and the need to form the most homogeneous study groups possible. It was interesting to see that performance declined in the Control group. It might have contributed to the significant difference between the two groups by the second survey. As the only difference between the program of the two groups was table tennis training, we may assume that it contributed at least to the maintenance of performance. The current academic year was underway during the study, which may have influenced the general activity of the students. However, more detailed and extended comparison (e.g., including completion of various specific surgical skill developing courses) is needed to clarify and optimize the effect of table tennis and other sports (separately or in combination) on fine skill performance. When we recruited volunteers, we considered equality as a fundamental issue. Furthermore, we cannot exclude the factor that the students who registered for this study were interested in manual fields of medicine or not. It is also not known how long the effects of training will last and to what level performance may decline without continued training. We do not know how other sports’ training programs would change the student’s fine motor skills. Answering these questions requires a further series of studies.

## 5. Conclusions

Table tennis has been identified as a sport that can be used to significantly improve arthroscopy and develop moderate catheter manipulation skills on simulators. Specific table tennis sport training program execution has a positive effect on the speed and economy of the arthroscopy and interventional cardiology operation simulation test results. High-fidelity simulation is useful for evaluating the result of the identified sport-based practical skills development methods. An intensive and easily feasible table tennis training program was effective in improving medical students’ manual skills and their performance on simulators. We continue the studies to further investigate various training programs and sports.

## Figures and Tables

**Table 1:** Selected anthropometric data.

	Control Group (n = 13)		Table Tennis Group (n = 13)	
	First survey	Second survey	First survey	Second survey
Male/Female	11/2		11/2	
Age	22 ± 1 years		22 ± 2 years	
BMI value	23.19 ± 2.34	22.89 ± 2.3	24.4 ± 4.48	23.47 ± 3.18
Right-handed/Left-handed	12/1		9/4	

**Table 2:** The time required to reach the given coronary vessels.

	Control Group (n = 13)		Table Tennis Group (n = 13)	
	First Survey (s)	Second Survey (s)	First Survey (s)	Second Survey (s)
Aortic root	11.29 ± 3.27	19.31 ± 7.5 *	14.38 ± 6.27	13.47 ± 5.9 ^#^
Left main	55 ± 21.64	95.54 ± 21.7 *	68 ± 28.16	87.4 ± 38.84
Left circumflex	183.93 ± 58.67	213.31 ± 55.43	176 ± 58.25	186.67 ± 57.54
Left anterior descendent	204.14 ± 68.75	244.92 ± 99.51	188.94 ± 52.39	193.53 ± 59.54
Right coronary artery	407.5 ± 82.81	453.38 ± 107.69	382.12 ± 90.38	373.13 ± 86.17 ^#^
Distal right coronary artery	442.43 ± 93.85	480 ± 106.87	408.19 ± 88.42	405.33 ± 83.51 ^#^

Means ± S.D., * *p* < 0.05 vs. First survey (paired *t*-test/Wilcoxon rank sum test); ^#^ *p* < 0.05 vs. Control group (Student’s *t*-test/Mann–Whitney rank sum test).

## Data Availability

The data presented in this study are available on request from the corresponding author. The data are not publicly available due to ethical permission constraints.

## Supplementary Materials

The following supporting information can be downloaded at https://www.mdpi.com/article/10.3390/s25175316/s1: Figure S1: Representative photos of completing tasks using the vascular catheterization simulator, and the arthroscopy simulator; Figure S2: A questionnaire filled in by the participants, including a consent form to participate in the research and use the data for scientific purposes; Table S1: Content of the 7-week table tennis training program.
