# Supplementary material for: An Innovative Application of High-Fidelity Medical Simulators to Objectively Demonstrate the Impact of Sports on the Development of Fine Motor Skills—A Pilot Study
Source: Sensors (Basel). 2025 Aug 27;25(17):5316. doi: 10.3390/s25175316 (PMC12430934; doi:10.3390/s25175316)
Supplement: Supplementary file 1 [file sensors-25-05316-s001.zip › sensors-3806940-supplementary.pdf]

Supplementary Materials: An Innovative Application of High-Fidelity Medical Simulators to Objectively Demonstrate the Impact of Sports on The Development of Fine Motor Skills – A Pilot Study

Peter Szikra, Adam Attila Matrai, Adam Varga, Laszlo Balogh, Zoltan Karacsonyi, Konrad Okros, Tamas Horovitz, Miklos Toth and Norbert Nemeth

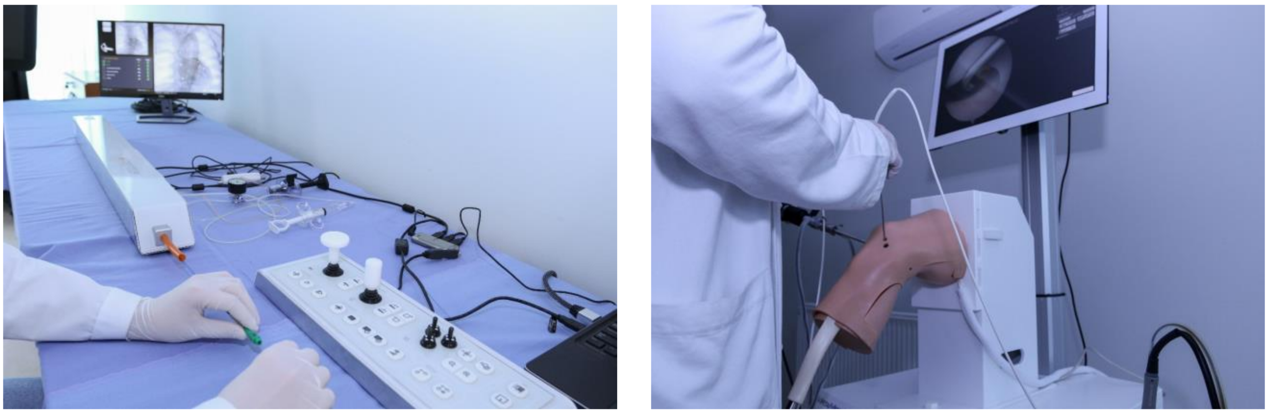

A **B**

Figure S1. Representative photos of completing tasks using (**A**) the vascular catheterization simulator and (**B**) the arthroscopy simulator.

Please fill in the questionnaire below! Thank you for your cooperation!

Name: ……………………………………………………...

Sex: ❐ male ❐ female **Age:** ……………

Anthropometers data:

bodyweight: ………kg height: ………… cm BMI-value: …………..

Right or left handed?
❐ right ❐ left

Do you play any instrument?
❐ yes ❐ no
If yes, what, when and how often? ……………………………………………………………..

Do you play video games?
❐ yes ❐ no
If yes, for how long and how often? ………………………………………………………………

How much do you use your (smart)phone each day?
❐ 2 hrs ❐ 4 hrs ❐ 6 hrs ❐ more

Do you do any arts and crafts hobby activities?
❐ yes ❐ no
If yes, what and when? ……………………………………………………………………………..

When does the survey take place?
❐ morning ❐ afternoon

Questions on physical activity:
- Do you do physical work? ❐ yes ❐ no
- What is your most common mode of transport? ❐ public transport ❐ car ❐ sport equipment ❐ walk
- Time spent sitting on average per day: ❐ 2 hrs ❐ 4 hrs ❐ 6 hrs ❐ more
- Do you do weight training? ❐ yes ❐ no

  If yes, with what weights and how often? ……………………………………………………..
- Do you do any other sporting activities?
  If yes, for how long and how often?

……………………………………………………..

……………………………………………………..

By completing the questionnaire, I agree that the data (anonymous) can be used for scientific purposes by the project's professional participants in the evaluation of the survey.

Date: ………………………………………….

Signature: ……………………………………...

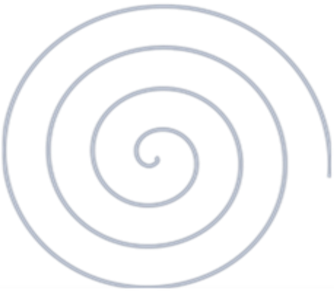

Follow the spiral line from the inside out without lifting the pen!

Figure S2. A questionnaire filled in by the participants, including a consent form to participate in the research and use the data for scientific purposes.

Table S1. Content of the 7-week table tennis training program.

Phase/Task	Task 1	Task 2	Task 3	Task 4	Task 5	Task 6	Task 7
**Week 1**	Starting position: racket in both hands, small straddle stance, arms in front of center position. Task: Touch the handle and the head of the rackets with a 180-degree wrist rotation. In the second series, the arms are held at an angle in the middle, and in the third series, they are held at an angle above the head.. Number of repetitions: 3x15	Starting position: racket in both hands, small straddle stance, arms in front of center position, ball between rackets in front-center position. Task: squat down with a straight back, touch the ground with the left racket. Stand up, squat down and touch the ground with the right racket. The ball cannot fall. Number of repetitions: 3x10	Starting position: racket in the dominant hand, pen handle grip. Task: read and say aloud the numbers shown by the partner while bouncing the ball. After half a minute, switch hands and person. Number of repetitions: 4x half a minute.	Starting position: small spread, normal racket grip. Task: bounce the ball on the racket in front of the body four to eight times, then bounce higher, turn around the axis of the body and continue. Number of repetitions: 4x1 minute with half-minute breaks, alternating hands (if difficult, then only with the dominant hand).	Starting position: sitting cross-legged, racket in both hands. Random bouncing of the ball on the rackets, playing from one to the other. The ball can bounce several times on one racket, the goal is to keep the ball in game, the ball cannot fall. Duration: 4 minutes.	Starting position in pairs facing each other 2 meters away, racket in hand with pen handle grip, small crosscut straddle stance. Task: after a few ball bounces, pass it to the partner, who also bounces the ball a few times and passes it back. Duration: 4 minutes	After completing the exercises (task 1-6), informal game of table tennis (every week).
**Week 2**	Change: squat down and stand up during the touchdown. There should be 2-3 touches during one squat.	Change: none	Change: with both hands, alternately	Change: try to achieve rotation around the axis without bounce the ball too high.	Change: the exercise starts in a kneeling position, while slowly sitting on the heels and rising back to kneeling (difficult).	Change: while moving, transfer the racket to the non-dominant hand, thus performing the exercise alternately with both hands.	
**Week 3**	Change: none	Change: touch the ground with the handle of the racket at every second bounce.	Change: none	Change: turn in both directions alternately	Change: Occasionally bounce the racket backwards and then back to the palm position.	Change: none	
**Week 4**	Change: none	Change: none	Change: none	Change: rotation after three bounces	Change: preferably the ball should only	Change: none	

					bounce once on the racket		
**Week 5**	Change: instead of squatting, the racket touches are performed while bending the trunk to the left and then to the right repeated twice.	Change: instead of squatting, kneel down and back	Change: none	Change: none	Change: none	Change: perform the task with the non-dominant hand	
**Week 6, 7**	Performing the exercises of week 4 and 5 alternately in each workout.	Change: none	Change: with both hands, alternately	Starting position: small spread, normal racket grip.<br>Task: bounce the ball on the racket in front of the body four to eight times, then bounce higher, turn around the axis of the body and continue. Number of repetitions: 4x1 minute with half-minute breaks, alternating hands (if difficult, then only with the dominant hand).	Starting position: sitting cross-legged, racket in both hands. Random bouncing of the ball on the rackets, playing from one to the other. The ball can bounce several times on one racket, the goal is to keep the game going for a long time.<br>Duration: 4 minutes.	Starting position in pairs facing each other 2 meters away, racket in hand with pen handle grip, small cross-legged landing.<br>Task: after a few bounces of the ball, pass it to the partner, who is also bounce the ball and passes it back.<br>Duration: 4 minutes	
